# Lessons Learned from Telemedicine in Adolescent Obesity: Results of a Pilot Study

**DOI:** 10.3390/children11050599

**Published:** 2024-05-16

**Authors:** Lenka Veselá, Aneta Klímová Rych, Anna Vážná, Markéta Kotrbatá, Kristina Rücklová, Irena Aldhoon-Hainerová

**Affiliations:** 1 Department of Children and Adolescents, Third Faculty of Medicine, Charles University and University Hospital Královské Vinohrady, 100 34 Prague, Czech Republic; lenka.vesela@fnkv.cz (L.V.);; 2 Regional Hospital Kolín, Hospital of Central Bohemian a.s., 280 02 Kolín, Czech Republic; 3 Department of Psychiatry, First Faculty of Medicine, Charles University and General University Hospital in Prague, 120 00 Prague, Czech Republic; 4 Department of Anthropology and Human Genetics, Faculty of Science, Charles University, 128 00 Prague, Czech Republic; 5 Central Laboratories, Department of Biochemistry, University Hospital Královské Vinohrady, 100 34 Prague, Czech Republic

**Keywords:** obesity, adolescents, telemedicine, cardiometabolic prevention, healthy lifestyle support, parental involvement, weight reduction, mental health

## Abstract

The rising prevalence of obesity in children calls for new strategies for the provision of effective care by a multidisciplinary team. Telemedicine has overall proven to be an effective tool for promoting a healthy lifestyle. The main objective of the current paper is to present the protocol of our ongoing CardioMetabolic Prevention (CAMP) study and compare its design with published studies on telemedicine in paediatric obesity. Additionally, we analysed the preliminary anthropometric and laboratory data to test the efficacy of our 12-week intensive program that combines in-person and telemedicine support. The program demonstrated a positive impact on body mass index (BMI) and its z-scores in 21 adolescents, and BMI in 18 participating parents. However, we found no effect on body composition, waist circumference, cardiometabolic parameters, or fitness evaluated via a 6-min walk test in adolescents. In conclusion, the combination of in-person and telemedicine intensive support over 35 h delivered by a multidisciplinary team can be beneficial not only for adolescents with obesity but also for their parents. The ongoing CAMP study serves as a platform for precision medicine in future decisions regarding anti-obesity medication in adolescents with obesity.

## 1. Introduction

Obesity is a chronic, relapsing, and multifactorial disease that is associated with adverse health and psychosocial consequences. According to the World Obesity Atlas, the prevalence of obesity is expected to rise sharply from 2020 to 2035, most notably among children and adolescents [1]. The rate is projected to increase from 10% to 20% among boys and from 8% to 18% among girls worldwide. It is widely acknowledged that up to 84% of children who suffer from obesity during childhood are likely to have a body mass index (BMI) above 30 kg/m^2^ in adulthood [2]. Recent studies have indicated that the COVID-19 pandemic has resulted in a significant rise in the prevalence of obesity [3], including among children, as evidenced by a study conducted on Czech children [4].

Childhood obesity is caused by a combination of genetic, environmental, and socioeconomic factors that affect children and their families. Obesity in children and adolescents can lead to various health risks, including dyslipidaemia, type 2 diabetes mellitus, metabolic dysfunction-associated fatty liver disease (previously known as non-alcoholic fatty liver disease), hypertension, obstructive sleep apnoea, polycystic ovary syndrome, musculoskeletal disorders, and various psychological problems [5]. The global economic impact of obesity, which includes both direct and indirect costs, is estimated to rise from 2.4% to 2.9% of the global gross domestic product by 2035 [1]. The World Health Organisation has recognized childhood obesity as one of the most serious public health challenges of the 21st century [6]. In many countries around the world, obesity in children and adolescents is one of the most common paediatric chronic diseases.

Childhood obesity is currently managed through lifestyle intervention, pharmacotherapy, and metabolic and bariatric surgery. It is widely known that even a modest weight loss of 5% can have a positive impact on cardiometabolic complications and is clinically beneficial [7,8]. Lifestyle intervention is the cornerstone of obesity management and should be aimed at the whole family. A multidisciplinary team is required to provide comprehensive obesity care that helps patients and their families to change their lifestyle, behavioural, and environmental factors. The team should include a paediatric healthcare provider, a nutritionist, a physiotherapist, an expert trained in cognitive behaviour therapy (CBT), and a psychologist. However, this approach to treating obesity in children is often time-consuming, expensive, poorly reimbursed, and may not be available in underserved regions and rural areas. Obesity management is associated with a high rate of non-compliance that can sometimes be due to high demand on time, money, and mobility [9]. Traditional lifestyle intervention programs, e.g., those delivered in hospitals, may not be suitable for every family [10]. 

Telemedicine can be used in various ways in the management and care of patients living with obesity. Firstly, remote monitoring can provide clinical data such as blood glucose, heart rate, sleep duration, level of physical activity, and energy intake. Modern technology can be used for data evaluation and data storage and can provide feedback to the patient. Secondly, telemedicine tools can serve as a communication resource between the patient, their family, and specialists. Finally, online support for physical activity can be effective. Young people are usually more receptive to using new technology as part of their treatment [11]. Several meta-analyses and reviews have investigated the effectiveness of telemedicine in reducing body weight in children and adolescents [12,13].

In the Czech Republic, there is currently no unified public healthcare platform that focuses on promoting and maintaining a healthy lifestyle in families with children living with obesity. Thus, there is an unmet need to identify effective strategies to address this issue. The main objective of this paper is to present the preliminary results of our study, which combines in-person and telemedicine approaches to promote healthy lifestyles in adolescents with obesity. Secondly, we discuss the efficacy of published studies on telemedicine in paediatric obesity to provide a sound background to our study protocol. 

## 2. Materials and Methods

### 2.1. Study Design

**Figure 1:**
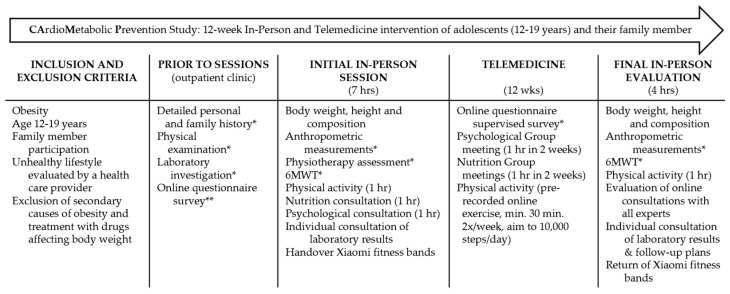
The protocol design of the pilot CardioMetabolic Project (CAMP) Study. Obesity is defined as body mass index ≥97th percentile for age and gender [14]. * only adolescents, ** only family members. In-person and telemedicine online sessions are separate group sessions for adolescents and family members. The anthropometric examination consists of the measurement of body circumferences and body skinfolds. Body composition is assessed according to body impedance using InBody 270. Abbreviations: 6MWT, six-minute walk test.

The CardioMetabolic Prevention (CAMP) study is an ongoing project that has been conducted by the Department of Children and Adolescents at the University Hospital Královské Vinohrady and the Third Faculty of Medicine at the Charles University in Prague, the Czech Republic since March 2022. The study aims to support healthy lifestyles among adolescents with obesity and their family members over 12 weeks, using a combination of in-patient and telemedicine approaches to address nutrition, physical activity, and psychological well-being (Figure 1). The study was approved by the Multicentric Ethics Committee of the University Hospital Královské Vinohrady on 2 March 2022, per the Helsinki Declaration II (EK-VP/05/0/2022). Additionally, it was registered with ClinicalTrials.gov on 27 April 2022 (NCT05350111). 

### 2.2. Study Population

Participants were recruited from new patients attending the outpatient obesity clinic. These patients underwent a physical examination, provided a detailed personal and family history, and shared information about their daily lifestyle. The study enrolled patients with obesity (BMI ≥ 97th percentile defined by the Czech references for BMI, specified for gender and age [14]) aged 12–19 years with at least one family member, regardless of their body weight status. This age group was selected based on the notion of similar mental, emotional, and social maturity levels to ensure homogeneity within the group. Additionally, adolescents possess adequate cognitive capacity to complete assessments, including questionnaire surveys. Exclusion criteria include secondary obesity due to endocrine and hereditary disorders evaluated by the paediatric endocrinologist and subjects treated with drugs affecting body weight. Since March 2022, the CAMP study has been offered to all patients who attend our outpatient obesity clinic and fulfil the study criteria. Before enrolment, all participants and their parent(s) signed their informed consent to participate in the study.

### 2.3. Study Outcomes

The main objective of the study was to evaluate the effectiveness of the CAMP study by examining changes in BMI, BMI standard deviation scores (z-score, z-BMI), body composition and laboratory cardiometabolic markers in adolescents, and changes in BMI and body composition in participating family members attending the program from March 2022 to June 2023. 

### 2.4. In-Person and Telemedicine Program

The CAMP study aims to educate and provide support to adolescents with obesity, along with at least one family member, in adopting a healthy lifestyle and to reduce body weight in all participants with excess body weight. The CAMP team is composed of two medical professionals, a clinical anthropologist, a physiotherapist, three psychotherapists, and two dietitians. Our multidisciplinary team conducts two rounds each year, and each round accommodates a group of 10–12 adolescents.

Both the in-person and the telemedicine sessions referred to in the current paper focused on all aspects of a healthy lifestyle, including nutrition, physical activity, and well-being. The sessions were held separately for groups of adolescents and their family members; they included a 7 h initial in-person group session, followed by 24 h of the telemedicine program over 12 weeks, and a final in-person evaluation for 4 h (Figure 1).

During the initial in-person session, all adolescents and their family members participated in an hour of aerobic physical activity. The physiotherapist screened the adolescents and evaluated their physical fitness using a 6 min walk test (6MWT). If necessary, a referral letter was provided for regular follow-up sessions with a physiotherapist. A separate group of adolescents and their family members attended an education session on healthy eating and received psychological support using CBT. Between March 2022 and June 2023, all participants, including the family members, received Xiaomi smart bands for 12 weeks. The bands helped them self-monitor their daily step count to reach an average of 8000–10,000 steps per day.

The 12-week telemedicine CAMP program consisted of online group consultations with both a nutritionist and a psychotherapist (Figure 1). There were six one-hour-long online consultations for each group (adolescent/family member) with psychotherapeutic support to increase motivation and discuss barriers to lifestyle changes and coping mechanisms. Another six hours were dedicated to nutrition by a dietitian who discussed the rules of healthy eating, including the plate system, home cooking, grocery shopping, reading nutrition labels, and snack preparation. The online consultations led by a nutritionist with adolescents focused on more practical aspects of nutrition, such as food choices in challenging circumstances (all-inclusive holidays, eating out) and buying snacks. Between March 2022 and June 2023, twice a week, there were new pre-recorded online exercises (30 min in length) led by a physiotherapist, which were freely available for both the adolescents and their family members. Compliance of all participants in online sessions was monitored. After 12 weeks, participants attended an in-person half-day weekend session for overall evaluation (Figure 1). A follow-up appointment in the outpatient clinic was offered to all participants 12 weeks later.

### 2.5. Studied Variables 

At the beginning and the end of the 12-week telemedicine program, the adolescent and a family member were asked to complete several questionnaires related to their lifestyle (screentime, dietary habits, physical activity, etc.), well-being, and mental health. These questionnaires included the Beck Depression Inventory [15], Eating Inventory [16], Rosenberg Self-Esteem Scale [17], Youth Eating Disorder Examination Questionnaire [18]/Eating Disorder Examination Questionnaire [19], Kidscreen-52 child/adolescent or parent version [20,21,22], Satisfaction with Life Scale child [23,24]/adult [25,26], and the State–Trait Anxiety Inventory for children/adults [27,28]. The questionnaires were administered by a psychologist or psychologist-trained professional who guided the children through the online process. Adults received instructions on how to complete the online questionnaires.

During the initial and final in-person education sessions, adolescents and their family members were measured. These measurements were taken in underwear and without shoes by a single clinical anthropologist using standard techniques [29]. Body height was measured to the nearest 0.5 cm using a calibrated wall-mounted stadiometer. Body weight and body composition (total body fat mass and fat-free mass) were assessed via bioimpedance using a InBody 270 (InBody Co., Seoul, Republic of Korea, DSM-BIA technology). The BMI was calculated by dividing body weight in kilograms by square height in meters. For adolescents, z-BMI was calculated with RůstCZ 2.3 software using data from the Czech children population as a reference [30]. Adolescents also underwent detailed anthropometric measurements of arm, waist, abdomen, and hip circumferences using a soft metric tape (0.1 cm). Skinfold thickness measurement was assessed with Best Caliper on selected sites (abdomen, above the iliac crest, triceps, biceps, and below the scapula) using standard procedures [31].

Laboratory investigations were conducted on adolescents before and after the 12-week program. All samples were collected after an overnight fast. Enzymatic assay with colour reactions (Siemens Atellica) was used to determine serum concentrations of uric acid, total cholesterol, and triglycerides. The concentrations of serum glucose and high-density lipoprotein cholesterol were determined via hexokinase reaction and homogenous enzymatic assay (Siemens Atellica), respectively. Liver enzymes were assessed spectrophotometrically using the Siemens Atellica analyser. The Friedewald formula was applied to calculate low-density lipoprotein cholesterol. Apolipoprotein A-I and apolipoprotein B were analysed via nephelometry (Siemens Atellica) and lipoprotein(a)was determined via turbidimetry (Roche Cobas Integra). Hormonal investigations (thyroid-stimulating hormone, free thyroxine, insulin, testosterone, oestradiol), 25-OH vitamin D, folic acid, homocysteine, and active vitamin B12 were assessed via the chemiluminescence method on the Siemens Atellica analyser. The homeostasis model assessment insulin-resistance index was calculated as fasting insulin x fasting blood glucose/22.5. The results of laboratory investigations were discussed by the medical team with each family individually at in-patient sessions.

### 2.6. Statistical Analysis

The pilot study’s preliminary data collected between March 2022 and June 2023 were analysed using the following methods. For descriptive analyses, mean and standard deviations (SD) were calculated for numerical variables. To assess the effectiveness of the 12-week intervention on adolescents and their family members, effect sizes and their corresponding 95% confidence intervals (CI) were calculated for the studied parameters before and after the intervention. In adolescents, the Wilcoxon signed-rank test was used to evaluate the difference in effect between z-BMI before and after the intervention. RStudio software (version 2023.03) was applied for all statistical analyses. 

## 3. Results

Preliminary data from the CAMP study conducted from March 2022 to June 2023 were acquired on three separate occasions (March to June 2022, October 2022 to January 2023, and March to June 2023); 65 families were offered study participation. Out of these, 30 adolescents and their parents took part in the program (46%). We present data from 21 adolescents (11 boys and 10 girls) with a mean age of 14.98 ± 2.45 years and 18 parents who completed the final evaluation and participated in all anthropometric and laboratory assessments (Table 1 and Table 2). Three families (10%) dropped out during the intensive program, and six families were unavailable for the final in-person session. 

The preliminary results demonstrated a positive impact of the intervention on BMI and z-BMI in adolescents and on body weight and BMI in parents. The difference in effect between z-BMI before and after the intervention was significant (*p* = 0.02). However, there was no significant change in waist circumference or the amount of total fat mass in either of the studied groups (Table 1 and Table 2). In total, 55% of adolescents decreased their z-BMI, 29% were able to stabilize their z-BMI, and 19% of adolescents increased their z-BMI. In the group of parents, 14 out of 18 (77.8%) decreased their BMI, while 2 out of 18 did not change their BMI and 2 out of 18 increased their BMI. Our preliminary analyses in adolescents did not show any effect on any of the studied laboratory parameters, some of which are presented in Table 1. Additionally, the 12-week intervention did not impact the results of the 6MWT performed by adolescents (Table 1). It was impossible to retrieve data on daily physical activity and steps from the fitness bands. Analyses of additional variables, such as the results of questionnaires, parent–child interactions, and compliance, will be conducted on a larger sample size in the future. 

## 4. Discussion

Our preliminary results indicate that the CAMP intervention has had a positive impact on the BMI and z-BMI of adolescents. A recent meta-analysis also demonstrated that various eHealth-based lifestyle strategies can be effective for the prevention and treatment of overweight and obesity in children and adolescents [12]. Those studies showed a significant reduction in BMI, z-BMI, waist circumference, body weight, and body fat percentage. In line with our findings, a pilot study by Fleischman et al. also found a significant reduction in BMI in children aged 10–17 years who participated in in-person appointments and 12 online consultations with a dietitian or psychologist for six months, even without active participation in physical activity [32]. Interestingly, some studies have concluded that the use of digital and technological tools in the management of children with obesity can lead to greater compliance among families [33]. Over the three rounds of our intensive CAMP program, there was a dropout of 10% of families (3/10) who had not participated already in the online sessions. Six families were unavailable for the final in-person session (e.g., due to illness) and thus, we did not include these families in the preliminary analyses. 

We have identified various responses to our intensive program ranging from body weight loss to stabilization or weight gain. It is widely recognized that not only body weight but also the response to weight reduction programs is genetically determined [34]. Furthermore, obesity as a complex and heterogeneous disease requires management based on individual stratification, which will also aid in the selection of anti-obesity treatment [35]. The CAMP study, therefore, forms an ongoing platform for precision-based medicine and future decisions. We have observed that adolescents who have been unable to lose weight despite high program adherence may benefit most from anti-obesity medication (personal observation).

We did not demonstrate any impact on body composition or fitness as assessed via the 6MWT. Body composition and fitness are linked and can be influenced by the level of physical activity. It is crucial to encourage physical activity and reduce sedentarism as an integral part of obesity prevention and management. Motion sensors can help in accurately documenting real physical activity in children and adolescents, as self-reported physical activity tends to be overestimated [36]. During our study, we urged adolescents and their parents to walk at least 8000–10,000 steps per day and participate in recorded online exercise sessions. We may speculate that solely online pre-recorded exercise sessions without supervision and monitoring of daily physical activity (including the number of daily steps) using fitness bands with no remote monitoring by the team were not sufficient. For the ongoing study, we now have smartwatches (Garmin Instinct 2S) that will allow the team to monitor participants remotely, including physical activity, sleeping patterns, and heart rhythm variation. Studies conducted on children have concluded that online exercise programs [36] or exergaming [37] are more effective than only monitoring tools. A recent study performed over six months also showed no significant effect on fat percentage in telehealth and on-site groups [38]. To improve both body composition and fitness, innovative physical activity options are sought to promote energy expenditure in individuals with obesity. 

Telemedicine in paediatric obesity provides different types of support related to its content, approach, and duration. Our CAMP study addresses complex aspects of a healthy lifestyle through utilizing CBT, which has been proven to be effective in telemedicine. Promising results have been demonstrated in a mobile-health pilot study reported by Pretlow et al., whose CBT-based strategy was applied to participants aged 8–20 years to address eating addiction, including binge eating, craving, and snacking [39]. Many telemedicine projects aim at reducing weight in children by promoting healthy eating through various methods. Several studies have demonstrated that telemedicine can positively impact the dietary habits of children and adolescents [40,41]. A study by Woolford et al. showed that personalized text messages sent via mobile phones, containing motivational texts, dietary information, and food recipes, may be helpful in conjunction with traditional care for adolescents with obesity [42]. A pilot study of a weight loss smartphone app, which involved coaching once a week, focused on avoiding snacking and reducing meal size, demonstrated high retention and adherence rates and resulted in a reduction of z-BMI in a more cost-effective manner than in-clinic intervention [43]. 

Frequent clinical visits have shown better outcomes in weight reduction, as shown by Look AHEAD [44] and other studies [45]. However, it can be challenging for patients and families to keep up with these appointments. To address this, the CAMP program offers 35 h of lifestyle support using both in-person and telemedicine approaches. A systematic review of 10 studies found that combining telehealth and face-to-face interventions led to improved outcomes in obesity treatment for children and adolescents [46]. In addition, studies have concluded that patients prefer online multidisciplinary support from familiar experts to manage their weight [47]. Combining telemedicine with in-person appointments has also been found to lead to greater weight reduction, as demonstrated by Alencar et al. [48]. It has been established that at least 26 contact hours are required to improve weight status in children with obesity, regardless of the mode of delivery [49]. However, Sela Peremen et al. found that the group with online support had a higher rate of adherence (up to 25%) to the program [38].

A recent meta-analysis has shown that parental or school engagement can greatly improve the effectiveness of health interventions [12]. An 8-month e-health lifestyle program that involved adolescents and their parents monitoring their dietary, physical activity, and sedentary behaviours through websites successfully altered the weight trajectory of adolescents with overweight or obesity [41]. A meta-analysis conducted in 2022 found that the combination of parent participation and intervention duration significantly predicted the effectiveness of the intervention. Another systematic review and meta-analysis revealed a significant decrease in BMI in the intervention group [13]. However, this significance was lost when parents were not involved in the program. Medium- to high-intensity involvement of parents/caregivers is essential and has been associated with greater effectiveness but also has led to improved family relationships and offspring motivation [50,51]. 

Our pilot study is unique because family members/parents of all bodyweight categories participated not only in the program but also in the assessment of body composition. A limited number of studies have reported on weighing parents as part of telehealth interventions. A 2-year interactive behavioural internet program showed significant bodyweight reduction in parents after the first 6 months but not after 2 years when compared with the control group [52]. The BMI of our parents ranged from 18 to 44 kg/m^2^, with a mean BMI of 29.3 kg/m^2^. Strikingly, the average body weight of our parents was significantly lower in comparison to their offspring (81.5 ± 16.4 kg vs. 102.3 ± 40.0 kg). After the COVID-19 pandemic, the number of children with severe degrees of obesity has increased dramatically [4]. As mentioned earlier, obesity has a strong genetic component and it would therefore be expected that family members are likely to be affected. However, in recent years, due to the obesogenic environment and unhealthy behaviour of youth, obesity may often affect the offspring of parents with a normal weight. Out of the 18 parents, BMI was in the category of normal weight, overweight, and obesity in 16.7%, 38.9%, and 44.4% of parents, respectively. For future analyses, we will study the interaction of BMI within parent–child pairs and the results of psychological evaluation. Based on our observations, a parent with normal/low weight and a child with obesity could indicate psychological issues within the family or child’s social environment, or the possibility of eating disorders.

Many studies on telemedicine in obesity have primarily focused on outcomes related to BMI, rather than laboratory cardiometabolic markers. While our pilot 12-week telemedicine intervention showed a positive effect on z-BMI (−3.2% decrease), no significant impact on the studied laboratory parameters was observed. Few studies have demonstrated a significant impact of telemedicine on serum cardiometabolic risk factors [53]. One study by Sela Peremen et al. reported a statistically significant change in haemoglobin A1c after a 6-month telehealth-based treatment program for children with obesity [38]. Another study that lasted 24 weeks, which provided exergaming and fitness coaching, improved systolic and diastolic blood pressure, total cholesterol, and low-density lipoprotein cholesterol in the intervention group [37]. Similarly positive effects, including improvements in glucose metabolism and anti-inflammatory cytokines, were observed after a year-long intervention for children between 2–18 years in all weight categories within the National e-Health Program for the Prevention and Management of Overweight and Obesity in Childhood and Adolescence in Greece [54]. As mentioned earlier, a weight loss of 5% in adults usually positively impacts cardiometabolic complications [7,8]. We may speculate that longer interventions, more pronounced weight loss, and increased physical activity may be necessary to achieve significant changes in laboratory parameters during adolescence. For future analyses, we also intend to study the effect on the cardiometabolic markers based on the baseline status of metabolically healthy or unhealthy obesity in a larger study cohort of adolescents [55,56]. 

We acknowledge that our pilot CAMP study has some limitations. During the pilot study, the lack of live online exercise sessions and the absence of devices for remotely monitoring physical activity (e.g., smartwatches) could have affected compliance and the results, especially regarding body composition. There was a rather high dropout from the pilot study, due to the unavailability of six of the families for the final in-person session. At the time of the pilot study, we were not permanently equipped with the bioimpedance device, which made it difficult to measure these families on alternate dates. To improve our study protocol, we now utilize smartwatches (Garmin Instinct 2 Solar, Garmin Ltd., Olathe, KS, USA) and are equipped with a bioimpedance device (InBody 270). Another limitation of the pilot study is the lack of a control group undergoing traditional outpatient follow-up visits for comparison. Lastly, we may speculate about the insufficient duration of our study, as most interventions lasting longer than 12 weeks have shown significant positive effects on BMI [12]. 

## 5. Conclusions

Our preliminary findings from the CAMP study have shown that combining in-person and telemedicine approaches with parental involvement and intensive contact with the multidisciplinary team is feasible and effective in addressing the challenges of providing health services to adolescents with obesity. We have found that including parents can have a positive impact on their body weight. Our analysis has revealed that relying solely on self-monitoring of physical activity is not sufficient. Consequently, we have modified our approach for the upcoming sessions by incorporating remote monitoring devices, continuous availability of bioimpedance, and live online exercise options. We are planning additional analyses to evaluate the influence of compliance, psychological screening results, and parent–child interaction on the study outcomes. The CAMP study is an important platform for tertiary obesity management care. We hope that the detailed database and the results of CAMP will help us in patient stratification and providing individualized precision-based medical care to these patients. Especially with the rising number of new anti-obesity agents, proper selection of patients who will profit the most from these therapies will be necessary.

## Figures and Tables

**Table 1:** Selected studied parameters in 21 adolescents before and after intervention and the analysis of the effects.

Parameter	Baseline Mean (SD)	Final Mean (SD)	Mean Effect (95% CI)
Body weight (kg)	102.3 (40.0)	101.4 (40.0)	0.90 (−0.4; 2.2)
BMI (kg/m^2^)	36.6 (10.9)	33.7 (7.9)	1.5 (0.2; 2.8)
z-BMI	3.1 (0.8)	3.0 (0.9)	0.1 (0.0; 0.2)
Total body fat mass (%)	43.4 (8.8)	41.8 (7.7)	1.56 (−0.0; 3.1)
Waist circumference (cm)	108.7 (23.8)	103.1 (19.8)	4.7 (−1.1; 10.4)
Blood glucose (mmol/L)	5.04 (0.3)	5.0 (0.4)	0.04 (−0.2; 0.2)
Insulin (mIU/L)	23.0 (16.9)	22.6 (13.9)	0.44 (−2.6; 3.4)
Uric acid (µmol/L)	376.9 (90.6)	378.0 (124.0)	8.86 (−12.6; 30.4)
HDL-C (mmol/L)	1.04 (0.2)	1.03 (0.3)	0.01 (−0.1; 0.1)
LDL-C (mmol/L)	2.78 (0.9)	2.57 (0.8)	0.21 (−0.1; 0.5)
TG (mmol/L)	1.44 (0.6)	1.3 (0.6)	0.14 (−0.1; 0.4)
ALT (µkat/L)	0.69 (0.9)	0.57 (0.7)	0.12 (−0.1; 0.3)
6MWT (m)	615.4 (65.8)	640.2 (60.0)	−13.0 (−36.0; 10.0)

Data are presented as means and standard deviations. Abbreviations: 6MWT, 6 min walk test; ALT, alanine aminotransferase; BMI, body mass index; CI, confidence interval; HDL-C, high-density lipoprotein cholesterol; LDL-C, low-density lipoprotein cholesterol; TG, triglycerides SD, standard deviation; z-BMI, z-score of BMI.

**Table 2:** Selected anthropometric parameters in 18 parents before and after the intervention and the analysis of the effect.

Parameter	Baseline Mean (SD)	Final Mean (SD)	Mean Effect (95% CI)
Body weight (kg)	81.5 (16.4)	80.2 (16.2)	1.54 (0.5; 2.5)
BMI (kg/m^2^)	29.3 (5.9)	28.4 (5.3)	0.7 (0.4; 1.1)
Total body fat mass (%)	34.3 (11.2)	32.5 (9.3)	1.1 (−1.4; 3.7)

Data are presented as means and standard deviations. Abbreviations: 6MWT, 6 min walk test; ALT, alanine aminotransferase; BMI, body mass index; CI, confidence interval; HDL-C, high-density lipoprotein cholesterol; LDL-C, low-density lipoprotein cholesterol; TG, triglycerides SD, standard deviation; z-BMI, z-score of BMI.

## Data Availability

The data presented in this study are available upon request from the corresponding author.
